# Selected biomarkers as predictive tools in testing efficacy of melatonin and coenzyme Q on propionic acid - induced neurotoxicity in rodent model of autism

**DOI:** 10.1186/1471-2202-15-34

**Published:** 2014-02-25

**Authors:** Mashael Al-Ghamdi, Laila Al-Ayadhi, Afaf El-Ansary

**Affiliations:** 1 Biochemistry Department, Science College, King Saud University, P.O box 22452, Zip code 11495 Riyadh, Saudi Arabia; 2 Autism Research and Treatment Center, Riyadh, Saudi Arabia; 3 Shaik AL-Amodi Autism Research Chair, King Saud University, Riyadh, Saudi Arabia; 4 Department of Physiology, Faculty of Medicine, King Saud University, Riyadh, Saudi Arabia; 5 Therapuetical Chemistry Department, National Research Centre, Dokki, Cairo, Egypt

**Keywords:** Propionic acid, Melatonin, Coenzyme Q, Serotonin, Gamma amino-butyric acid, Dopamine, Oxytocin, Interferon γ-inducible protein 16, Comet DNA, Autism

## Abstract

**Background:**

Exposures to environmental toxins are now thought to contribute to the development of autism spectrum disorder. Propionic acid (PA) found as a metabolic product of gut bacteria has been reported to mimic/mediate the neurotoxic effects of autism. Results from animal studies may guide investigations on human populations toward identifying environmental contaminants that produce or drugs that protect from neurotoxicity. Forty-eight young male Western Albino rats were used in the present study. They were grouped into six equal groups 8 rats each. The first group received a neurotoxic dose of buffered PA (250 mg/Kg body weight/day for 3 consecutive days). The second group received only phosphate buffered saline (control group). The third and fourth groups were intoxicated with PA as described above followed by treatment with either coenzyme Q (4.5 mg/kg body weight) or melatonin (10 mg/kg body weight) for one week (therapeutically treated groups). The fifth and sixth groups were administered both compounds for one week prior to PA (protected groups). Heat shock protein70 (Hsp70), Gamma amino-butyric acid (GABA), serotonin, dopamine, oxytocin and interferon γ-inducible protein 16 together with Comet DNA assay were measured in brain tissues of the six studied groups.

**Results:**

The obtained data showed that PA caused multiple signs of brain toxicity revealed in depletion of GABA, serotonin, and dopamine, are which important neurotransmitters that reflect brain function, interferon γ-inducible protein 16 and oxytocin. A high significant increase in tail length, tail DNA% damage and tail moment was reported indicating the genotoxic effect of PA. Administration of melatonin or coenzyme Q showed both protective and therapeutic effects on PA–treated rats demonstrated in a remarkable amelioration of most of the measured parameters.

**Conclusion:**

In conclusion, melatonin and coenzyme Q have potential protective and restorative effects against PA-induced brain injury, confirmed by improvement in biochemical markers and DNA double strand breaks.

## Background

Autism is a neurodevelopmental disorder showing impairment in language, social interaction, repetitive and disordered movements [1]. Hyperactivity, sensory disturbances, and sometimes self injury are also observed signs [2,3]. Propionic acid (PA) occurs naturally in a few food products as milk although relatively higher concentrations are present in dairy products such as yogurt and cheese obviously due to bacterial fermentation [1,2]. The dietary sources however have a minor contribution in the PA levels in the body [3]. In the colon, PA is produced by fermentation of polysaccharides, oligosaccharides, long-chain fatty acids, protein, peptides and glycoprotein precursors by the anaerobic colonic microbiota, Undigested carbohydrates, such as dietary fibers and resistant starch, represent the major source for PA production [3]. PA is a short chain fatty acid formed endogenously in the human body as an intermediate of fatty acid metabolism and a metabolic end product of enteric gut microbiota such as clostridia and propionibacteria [3].

Interestingly, MacFabe et al. [4], through intraventricular infusions of PA, were able to induce behavioural and brain abnormalities in rats similar to those seen in humans suffering from autism via probably altering brain fatty acid metabolism [5-7]. Because large amounts of PA have been used by MacFabe et al. [4] to induce the symptoms (e.g. 4 μl of 0.26 M solution), it therefore remains to be answered, if PA produced by the microbiota could lead to similar effects. In a trial to answer this question, and to highlight the importance of gut-brain axis in the etiology of autistic features in rat pups, El-Ansary et al. [8] compared the neurotoxic effects of orally administered PA (250 mg/kg/day for 3 days) to those produced through induction of PA producers using clindamycin antibiotic. They declared that the neurotoxic effect of imbalanced gut microbiota in clindamycin-treated rats was much less than that of direct orally administered PA. There is also some evidence that high levels of PA can induce oxidative stress and glutathione depletion in various brain regions such as cortex, hippocampus, thalamus, and striatum of rats infused with PA (intraventricular infusions of 4 μl 0.26 M PA per animal. Catalase activity decreased in most brain regions suggesting a reduced antioxidant enzymatic activity [9]. High levels of subcutaneously added PA in rats (around 1.5–2 μmol/kg (body weight) caused slight but significant delays in the day of appearance of hair coat and eye opening, indicating an effect of PA on the development of physical parameters [10], which suggests that early postnatal PA administration to rats alters normal development and induces long-term behavioural deficits.

Brain tissue is very vulnerable to free radical damage because of its high oxygen utilization, high concentrations of polyunsaturated fatty acids [11] and transition metals such as iron, which is involved in the generation of the hydroxyl radical [12], and low concentrations of cytosolic antioxidants [13]. Glutathione (GSH) is the predominant antioxidant in the brain that is present at millimolar concentrations [14,15].

Recently, PA mechanism of action was clarified. It possesses many neuropharmacological oxidative properties which could be related to behavioral abnormalities seen either clinically in patients with autism or in rodent model of autism [13]. PA through oxidative mechanisms inhibits Na^+^/K^+^ ATPase [16] and increases glutamate receptor sensitivity which can enhance neural depolarization leading to neural hyperexcitability in brain regions linked to locomotor activity. It also promotes intracellular calcium release which is known to play a key role in synaptic transmission [17,18].

Melatonin possesses an electron-rich aromatic indole ring and functions as an electron-donor, thereby reducing and repairing electrophilic radicals [19]. Of additional interest regarding melatonin, is its reported to bind to quinone reductase 2 [20]. This enzyme, which is considered a melatonin receptor, is important in the detoxification of pro-oxidant quinones. Experimental evidence supports its actions as an indirect antioxidant when stimulating antioxidant enzymes [19], its ability to enhance the activities of other antioxidants and its protection of antioxidant enzymes from oxidative damage [20]. Several groups have shown that melatonin reverses chronic and acute inflammation [21,22]. Melatonin treatment also causes an important reduction of nitric oxide (NO) and malondialdehyde (MDA) levels, two compounds that are closely related to inflammation [23].

Mitochondrial dysfunction has been well established to occur and play an important role in the pathogenesis of autism [24]. A key component of the mitochondrial electron transport chain (ETC.) is coenzyme Q, which not only serves as the electron acceptor for complexes I and II of the ETC. but is also an antioxidant [25-27]. In addition to being crucial to the bioenergetics of the cell, mitochondria play a central role in apoptotic cell death through a number of mechanisms, and coenzyme Q can affect certain of these processes. Reduced coenzyme Q levels with subsequent increased free radical generation, reduced free radical scavenging and defective apoptosis leading to abnormal synaptogenesis were reported in autism [28].

This information motivates our interest to test the protective and therapeutic effects against PA-induced biochemical persistent autistic features in rat pups. Among the measured parameters, gamma amino butyric acid (GABA), serotonin and dopamine represent markers of brain chemistry, while heat shock protein 70 (Hsp70) and interferon γ inducible protein 16 were selected as inflammatory related parameters.

## Methods

### Animals

This is an interventional experimental animal study performed on forty eight inbred male western albino rats (45 to 60 g, approximately 21 days old). Rats were obtained from the Pharmacy College animal house at King Saud University. They were kept under standard conditions of temperature, 12-h dark/light cycle and were given free access to tap water and standard laboratory chow. After one week of acclimation, the rats were divided into six groups (eight rats in each group), namely the control group in which animals were fed normal diet during the experimental period; the PA treated rats that received 250 mg/kg body weight/day for 3 days, in order to induce autistic features; The third and fourth groups were treated with low dose of either coenzyme Q (4.5 mg/kg body weight) [29] or melatonin (10 mg/kg body weight) [30] for one week after being intoxicated with the PA as described above (therapeutically treated groups). Fifth and sixth groups were treated with either coenzyme Q or melatonin for one week followed by PA intoxication (protected groups). All groups of rats were housed under controlled temperature (21 ± 1°C) with ad libitum access to food and water. PA, melatonin or coenzyme Q were orally dosed to rat pups using gastric tube.

### Tissue preparation

At the end of the feeding trials, the rats were anesthetized with carbon dioxide and decapitated. The whole brain was removed from the skull and dissected into small pieces and homogenized in 10 times w/v bi-distilled water and kept at–80°C until further use for different biochemical analyses. A small piece of brain was kept separately for Comet DNA assay.

### Ethics approval and consent

This work was approved by the Ethical committee of Science College at King Saud University (Approval no 8/25/220358).

#### *Assay of heat shock protein 70 (Hsp70)*

HSP70 was measured in brain cortex and medulla homogenate using an ELISA kit, product of Uscn Life Science Inc. Wuhan, China according to the manufacturer’s instructions. The minimum detectable level of rat HSP70 detected is less than 0.045 ng/ml.

#### *Assay of gamma amino-butyric acid (GABA)*

GABA was quantitatively determined using the ELISA immunoassay kit from ALPCO Diagnostics (Salem, NH, USA). Least detectable amount is 7.5 ng/ml.

#### *Assay of serotonin*

Serotonin was measured using an ELISA kit from Immuno-Biological Laboratories (IBL, Hamburg, Germany). Brain homogenate preparation (derivatization of serotonin to N-acyl-serotonin) was part of the sample dilution which was achieved by the incubation of the respective sample with the acylating reagent. The assay procedure followed the competitive ELISA protocols whereby competition takes place between biotinylated and non-biotinylated antigens for a fixed number of antibody binding sites. The amount of biotinylated antigens bound to the antibodies was inversely proportional to the N-acyl-serotonin concentration of the sample. Analytical sensitivity of this product is 0.014 ng/ml.

#### *Dopamine assay*

Dopamine was extracted by using a cis-diol-specific affinity gel, acylated and then derivatized enzymatically. Quantitavive assay was performed using an ELISA kit, a product of Immuno Biological Laboratories (IBL) with detectable range of 12–2250 ng/ml.

#### *Assay of interferon μ inducible protein 16*

IFI16 was measured using ELISA kit a product of My Biosource applying the competitive enzyme immunoassay technique utilizing a monoclonal anti-IFI16 antibody and IFI16-HRP conjugate. The detectable range of this product is 23.5-1500 pg/ml with minimum detectable level of 5.9 pg/ml.

#### *Comet DNA assay*

Single cell gel electrophoresis or Comet assay is one of the simple, sensitive and rapid methods for the detection and quantification of DNA damage [31]. Slides were prepared in duplicate per group and the test was performed for at least 3 different brain samples from each group. Cell suspension, about 4 × 10^6^ cells were mixed with 80 μL of 0.7% low-melting agarose in phosphate-buffered saline (PBS) at 37°C in a microtube, and then spread over a window microscopic slide. The slides were pre-coated with 150 μL of 0.5% normal-melting agarose in PBS, and were specially designed for this assay. Then, slides were placed immediately in cold lysis buffer, 2.5 M sodium chloride NaCl, 100 mM EDTA sodium salt Na_2_EDTA, 10 mM Tris (pH 10), and 1% Triton X-100, at 4°C for a minimal of 1 hr. After lysis, the slides were drained and placed in a horizontal gel electrophoresis tank surrounded by ice, and filled with fresh cold electrophoresis buffer (300 mM sodium hydroxide NaOH, 1 mM NaEDTA, pH 13). To allow DNA unwinding, the slides were kept in the high pH buffer for 20 min. After that, electrophoresis was carried out for 20 min at 25 V and 300 mA. The slides were then drained and flooded slowly with 3 changes of neutralization buffer (0.4 M Tris, pH 7.5) for 5 min each, and then stained with 30 mL of ethidium bromide (20 mg/L), and covered with cover slips. All those steps were performed under dimmed light in order to prevent additional DNA damage caused by visible light. A total of 50 randomly selected cells per slide were analysed. Imaging was done using a fluorescence microscope (Zeiss Axiovert L410 Inc., Jena, Germany), attached to a digital camera (Olympus Inc., Tokyo, Japan), and equipped with 549 nm excitation filter, 590 nm barrier filter, and a 100-W mercury lamp. The percentage of DNA in the comet tail “DNA damage” was automatically calculated.

Comets were randomly captured at a constant depth of the gel, avoiding the edges of the gel, occasional dead cells, and superimposed comets. DNA damage was measured as tail length (TL = distance of DNA migration from the centre of the body of the nuclear core), and tail intensity DNA (TI =% of genomic DNA that migrated during the electrophoresis from the nuclear core to the tail). By presenting all three parameters together, more information could be obtained on the extent of DNA damage [29].

#### *Statistical analysis*

The data were analysed using the statistical package for the social sciences (SPSS, Chicago, IL, USA). The results were expressed as mean ± standard error of the mean (SEM). All statistical comparisons between the control and PA-treated rat groups were performed using the one-way analysis of variance (ANOVA) test complemented with the Dunnett’s test for multiple comparisons. Significance was assigned at the level of *P* <0.05. Receiver operating characteristics (ROC) curve analysis was performed. Area under the curve (AUC), cut-off values, and degree of specificity and sensitivity were calculated.

## Results

**Figure 1:**
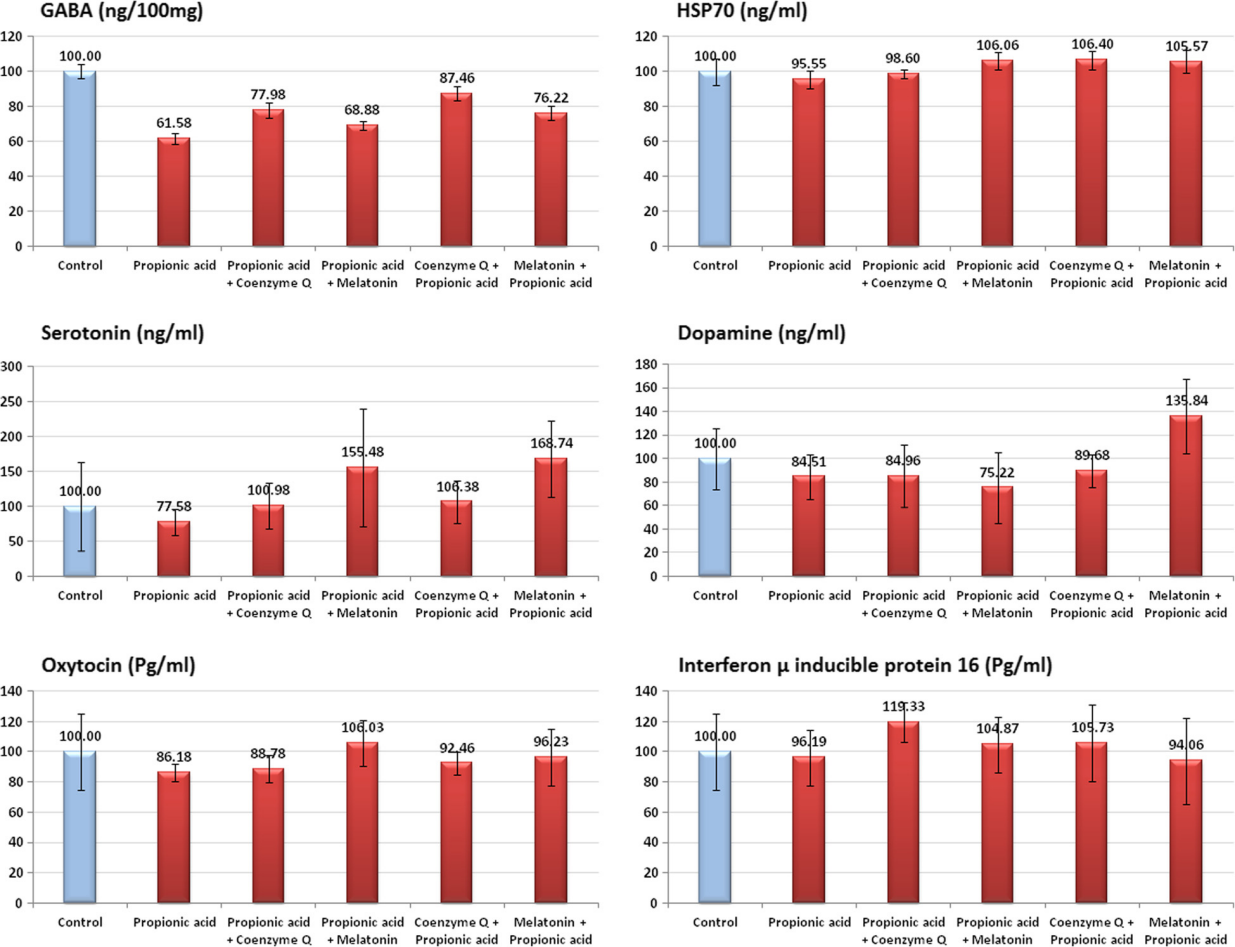
Percentage change ± S.D error bars of Hsp70, GABA, serotonin, dopamine, oxytocin, and IFI16 in PA-neurointoxicated, coenzyme Q and melatonin protected or treated rat pups compared to healthy control.

Tables 1 and 2 demonstrate data obtained as of the six measured parameters in the PA-treated, coenzyme Q or melatonin treated groups pre or post PA intoxication respectively compared to control. It was noticed that PA induced remarkable decrease in all parameters. GABA and serotonin were the most affected parameters, recording 38.42 and 22.48 percentage decrease respectively while Dopamine and oxytocin showed relatively less impaired levels (15.59 and 13.82% respectively). Hsp70 and IPI16 were the least affected parameters recording less than 10% reduced concentrations. PA treatment along with coenzyme Q or melatonin resulted in significant increase in the different parameters revealing a potent protective and therapeutic effect of both treatments when compared to PA-treated group. Figure 1 represents the percentage change of the measured parameters in treated groups compared to control.

**Table 1:** Mean ± S.D and independent t-test for HSP-70, GABA, serotonin, dopamine, oxytocin, and Interferon μ inducible protein 16 (IFI16) in neurointoxicated, Co Q protected and treated rat pups compared to healthy control

**Parameters**	**Group**	**N**	**Mean ± S.D.**	**P value**
**HSP70 (ng/ml)**	Control	8	10.563 ± 0.370	
	PA	8	9.400 ± 0.490	0.001^a^
	CoQ-Protected group	6	10.050 ± 0.404	0.030^a^
	CoQ-treated group	8	10.033 ± 0.427	0.029^a^
**GABA (ng/10 mg)**	Control	8	109.100 ± 4.666	
	PA	8	67.183 ± 3.125	0.001^a^
	CoQ-Protected group	6	95.417 ± 4.529	0.01^a^
	CoQ-treated group	8	85.079 ± 4.832	0.01^a^
**Serotonin (ng/ml)**	Control	8	0.073 ± 0.017	
	PA	8	0.047 ± 0.011	0.004^a^
	CoQ-Protected group	6	0.095 ± 0.018	0.043^a^
	CoQ-treated group	8	0.097 ± 0.018	0.033^a^
**Dopamine (ng/ml)**	Control	8	136.250 ± 18.468	
	PA	8	106.875 ± 12.229	0.002^a^
	CoQ-Protected group	6	169.167 ± 24.580	0.014^a^
	CoQ-treated group	8	160.000 ± 22.583	0.051^a^
**Oxytocin (Pg/ml)**	Control	8	89.750 ± 4.464	
	PA	8	84.500 ± 4.751	0.039^a^
	CoQ-Protected group	6	95.667 ± 4.967	0.037^a^
	CoQ-treated group	8	95.000 ± 3.742	0.038^a^
**Interferon μ inducible protein 16 (Pg/ml)**	Control	8	833.750 ± 63.906	
	PA	8	768.750 ± 53.033	0.044^a^
	CoQ-Protected group	6	931.667 ± 77.567	0.024^a^
	CoQ-treated group	8	983.333 ± 60.222	0.001^a^

^a^Significant level between the studied groups compared to control when P < 0.05.

**Table 2:** Mean ± S.D and independent t-test for HSP-70, GABA, serotonin, dopamine, oxytocin, and Interferon μ inducible protein 16 (IFI-16) in neurointoxicated, melatonin protected and treated rat pups compared to healthy control

**Parameters**	**Group**	**N**	**Mean ± S.D.**	**P value**
**HSP70 (ng/ml)**	Control	8	10.563 ± 0.370	
	PA	8	9.400 ± 0.490	0.001^a^
	Melatonin-protected group	8	10.043 ± 0.454	0.030^a^
	Melatonin-treated group	8	10.083 ± 0.445	0.048^a^
**GABA (ng/10 mg)**	Control	8	109.100 ± 4.666	
	PA	8	67.183 ± 3.125	0.001^a^
	Melatonin-protected group	8	83.160 ± 4.668	0.01^a^
	Melatonin-treated group	8	75.144 ± 2.694	0.001^a^
**Serotonin (ng/ml)**	Control	8	0.073 ± 0.017	
	PA	8	0.047 ± 0.011	0.004^a^
	Melatonin-protected group	8	0.101 ± 0.019	0.011^a^
	Melatonin-treated group	8	0.099 ± 0.019	0.016^a^
**Dopamine (ng/ml)**	Control	8	136.250 ± 18.468	
	PA	8	106.875 ± 12.229	0.002^a^
	Melatonin-protected group	8	173.125 ± 29.873	0.010^a^
	Melatonin-treated group	8	164.375 ± 23.213	0.018^a^
**Oxytocin (Pg/ml)**	Control	8	89.750 ± 4.464	
	PA	8	84.500 ± 4.751	0.039^a^
	Melatonin-protected group	8	97.750 ± 8.714	0.037^a^
	Melatonin-treated group	8	100.750 ± 6.409	0.001^a^
**Interferon μ inducible protein 16 (Pg/ml)**	Control	8	833.750 ± 63.906	
	PA	8	768.750 ± 53.033	0.044^a^
	Melatonin-protected group	8	921.250 ± 71.801	0.022^a^
	Melatonin-treated group	8	922.500 ± 79.057	0.027^a^

^a^Significant level between the studied groups compared to control when P **<** 0.05.

**Figure 2:**
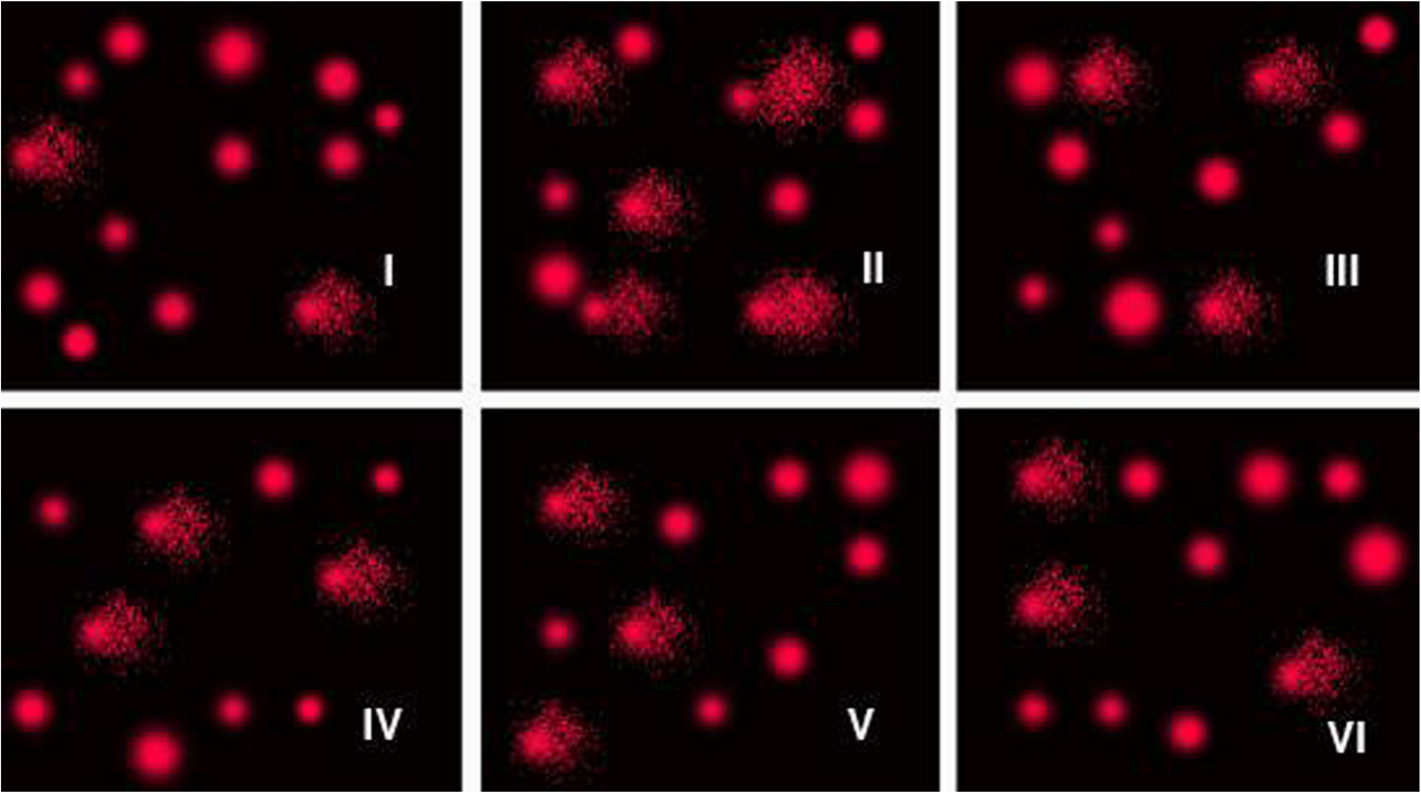
Photograph showing comet tailing in PA treated (II) together with the protective and therapeutic effects of melatonin and Co Q (III-VI) in rat brains compared to control (I).

Table 3 and Figure 2 demonstrate PA- induced DNA damage in the brain of treated rats as evident from the significant increase in the comet parameters, namely tail length (μm), tail DNA (%) and tail moment (arbitrary units). The table also demonstrates the potency of coenzyme Q or melatonin in protecting against and treating PA neurotoxicity. Both ameliorate the DNA damaging effects of PA observed as a significant decrease of PA-induced DNA damage. ROC analysis showed satisfactory values of area under the curve, sensitivity and specificity.

**Table 3:** Tail Length (μm), Tail DNA% and Tail Moments in PA-intoxicated, Co Q or melatonin supplemented compared to control- healthy rat pups

**Parameters**	**Groups**	**Min.**	**Max.**	**Mean ± S.D.**	**P value**
Tail Length (μm)	Control	1.06	1.42	1.25 ± 0.16	0.001
	PA	4.67	5.34	4.96 ± 0.28^a^	
	Melatonin-protected	3.17	4.33	3.56 ± 0.55^a^	
	Melatonin-treated	3.95	4.26	4.11 ± 0.13^a^	
	CoQ_10_-protected	2.87	3.05	2.94 ± 0.08^a^	
	CoQ_10-_treated	3.59	3.95	3.82 ± 0.16^a^	
Tail DNA%	Control	1.22	1.62	1.40 ± 0.17	0.001
	PA	4.51	5.15	4.85 ± 0.27^a^	
	Melatonin-protected	3.12	3.33	3.20 ± 0.09^a^	
	Melatonin-treated	3.52	4.26	3.95 ± 0.33^a^	
	CoQ_10_-protected	2.80	3.41	3.10 ± 0.25^a^	
	CoQ_10_-treated	3.16	3.75	3.55 ± 0.28^a^	
Tail Moments (Units)	Control	1.42	2.15	1.76 ± 0.37	0.001
	PA	21.96	25.78	24.07 ± 1.87^a^	
	Melatonin-protected	10.18	13.67	11.39 ± 1.57^a^	
	Melatonin-treated	15.00	17.79	16.20 ± 1.21^a^	
	CoQ_10_-protected	8.09	9.81	9.11 ± 0.76^a^	
	CoQ_10_-treated	11.33	14.83	13.58 ± 1.59^a^	

Table 3 describes the One-way ANOVA test between the Control, PPA, Melatonine + PA, PA + Melatonine, CO Q + PA and PA + CO Q groups in Tail Length (μm), Tail DNA% and Tail Moments (Units) groups and Dunnett test as multiple comparisons.

^a^Significant level between the studied groups compared to control when P < 0.05.

## Discussion

Animal models are helpful in understanding the mechanism and environmental factors which trigger the disease process. Studies of MacFabe et al., [9] have demonstrated that PA intraventricularly infused to rats provides a suitable animal model to study ASD. Moreover, there are a number of inherited and acquired conditions which lead to elevations of PA and these are related to developmental delay, seizures and gastrointestinal symptoms, resembling some aspects of autism. Thus, PA may be a putative link between dietary or enterobacterially derived metabolites along with genetic predisposition and subsequent features of autism [9,32-35]. As oxidative stress has been suggested as the primary mechanism of PA neurotoxicity, it was of great interest to test the protective and therapeutic effects of CoQ and melatonin as two supplements displaying antioxidant and free radical scavenger properties.

Hsp is generally much more sensitive to stress than other health indices, making its use as biomarker very common in toxicology [36]. Table 1 demonstrates that PA–treated rats show lower level of Hsp70 which is not in good agreement with our previous study in which the same PA dose induced remarkable elevation of this stress-induced protein. This could be attributed to the fact that potential mechanisms of stress tolerance differ among individuals, populations, and species [37]. This intra-specific variation might be due to genetic variation among individuals, nutritional status, reproductive status, antioxidant status of the individuals, Hsp mRNA stability and pre-existing pool of HSF (heat shock transcription factor) [38]. In the present study, inbred rats were used and led to the absence of intra-subject variability seen as low standard deviation (SD), (Table 1). The protective and therapeutic effects of melatonin shown in Table 1 could be supported by the work of Rodella et al. [39] in which they reported that melatonin was effective in increasing the reduced level of Hsp70 induced by nicotine in heavy smokers.

Undoubtedly, the magnitude and potential severity of many neurotoxic agents could be measured through the consequent changes in neurotransmitters as biomarkers of brain damage [13]. Table 1 demonstrates the remarkable lower levels of GABA, serotonin and dopamine in PA-treated rat brain homogenates compared to controls. This could be supported by the fact that, PA being capable to access to the brain could induce neurochemical effects on CNS function [8] including neurotransmitter synthesis and release. Of interest, PA is capable of altering dopamine, serotonin, GABA and glutamate systems in a manner similar to that observed in ASDs [40,13], partly via changing intracellular calcium release rate [41]. It could be easily observed that PA pre or post-treatment with Co Q (Table 1) or melatonin (Table 2) induced satisfactory amelioration of GABA levels, with Co Q being more potent compared to melatonin. Significant increased levels of serotonin and dopamine, even higher than control subjects was recorded by both supplements. This is consistent with the previous finding of Binukumar et al. [42] that pretreatment with Co Q caused a significant attenuation of the loss of striatal dopamine and dopaminergic neurons caused by the pesticide, dichlorvos through the activation of the mitochondrial respiratory chain reactions and mitochondrial antioxidant enzyme function. The protective potency of Co Q against brain neurotransmitters depletion as biochemical autistic features in PA-treated rat pups, could be related to the recent work of Kałużna-Czaplińska [43] in which he reported elevated succinic acid excretion as a marker of Co Q deficiency in children with autism.

Cardinali et al. [44] proved that preincubation of synaptosome-rich homogenates of rat hypothalamus with melatonin induced significant increases of norepinephrine, serotonin, dopamine and glutamate concentrations. They suggest that exogenously-administered melatonin may affect neurotransmitter accumulation and release in the hypothalamus by modification of the transmitter uptake mechanism rather than by competition with the transmitter for its uptake pump. This can help to understand the protective and therapeutic effects of melatonin reported in the present study and shown as remarkable elevation of serotonin and dopamine concentration.

It is worth noting that OT has been involved in the etiology of autism, with a sex-related pattern and hence it could be used as biomarker of PA-induced autistic features in rodent model [45]. Tables 1 and 2 demonstrate oxytocin levels in brain of control, PA, Co Q or melatonin-treated rat pups. It could be easily noticed that PA induce oxytocin depletion while both Co Q and melatonin were potent in ameliorating the neurotoxic effect of PA to a great extent. Depleted oxytocin could be easily correlated to the recorded depletion in GABA level shown in the same table. Endogenous oxytocin additionally functions as an anxiolytic, acting to increase release of the inhibitory neurotransmitter GABA in the central amygdale [46]. Cumulative evidence from rodent models suggests that both acute and chronic administration of oxytocin reduces physiological and behavioral stress responsively [47,48].

Inflammation in the nervous system is widely recognized as contributing to a number of neurological conditions. However, the central nervous system (CNS) has also been classically recognized as occupying a privileged site with respect to immune-related phenomena. This dichotomy is widely understood to be a functional manifestation of known CNS. The most prominent element involved in these mechanisms is the blood–brain barrier (BBB), a physical and metabolic barrier separating the CNS from the systemic circulation, creating a unique and stable environment for neuronal activity. Table 1 demonstrates that PA induced lower level of IFI 16 compared to control untreated rat pups. This is unexpected because Gariano et al. [49] pointed out that there is a signaling pathway linking oxidative stress as an aspect of PA neurotoxicity to upregulation of interferon (IFN)-inducible gene IFI16. The recorded low level could be related to the early finding of Dawson et al. 1998) [50] that IFI 16 is expressed in CD34+ and monocytoid daughter cells, but is rapidly and markedly down-regulated at the corresponding stages of polymorphonuclear and erythroid development. Moreover, glucose restriction in cells usually accompanied by high AMP/ATP ratio (energetic stress), which activates the AMPK/p53 pathway. Depending upon the energetic stress levels, cells undergo either autophagy or cell death. Given that the activated p53 induces the expression of IFI16 protein, Duan et al. [51] investigated the potential role of the IFI16 protein in glucose restriction-induced responses. Lower, IFI 16 protein reported in the present study could be related to the neurotoxic effect of PA making brain cells of treated rat pups less adapted to the energetic stress as a well known phenomenon of PA neurotoxicity. Additionally, the recorded low IFI 16 could be easily related to the pro-apoptotic effect of PA. It is well known that Interferons (IFNs) are multifunctional cytokines with antiviral, anti-proliferative and immunomodulatory effects. Activation of type-I IFN-signaling in immune cells inhibits the production of proinflammatory cytokines and activates inflammasomes. Given that the IFI16 and AIM2 proteins are IFN-inducible and can heterodimerize with each other, Veeranki et al., [52] investigated the regulation of IFI16, AIM2, and inflammasome proteins by type-I and type-II IFNs and explored that expression of IFI16 protein in THP-1 cells suppresses the activation of caspase-1. This explanation could be supported through considering the work of El-Ansary et al. [13] in which they recorded activation of caspase 3 as pro-apoptotic biomarker in brain tissue homogenates of PA-treated rat pups. The ameliorating effects of CoQ_10_ may be attributed to its role in activating the electron transport chain and hence make brain cells more adapted to energy depletion induced by PA. In addition, both CoQ_10_ and melatonin could be easily related to their antioxidant effects.

The results provide evidence that early PA treatment induces long-lasting behavioral deficits, which are possibly caused by oxygen reactive species generation, and suggest that oxidative stress may be involved in the neuropathology of propionic acidemia.

Table 3 and Figure 1 show that the comet assay is able to demonstrate the aetiology of DNA damage, induced by PA. This could be supported by certain previous studies which have shown that PA can increase ROS generation and oxidative damage to cells [13]. This hypothesis is reinforced by the work of McLaughlin et al. [53] demonstrating that exposure of striatal and cortical cultures from embryonic rat brain to PA for 24 h provoked DNA laddering and dose-dependent cell death, which was attenuated by antioxidants. PA stimulates lipid peroxidation in rat brain and in the plasma of patients with PAemia [54,55]. The recorded protective effect of Co Q shown in Table 1 could be supported by Papucci et al. [56] who reported that Co Q is able to counteract mitochondrial membrane potential depolarization, ATP depletion, cytochrome c release, caspase-9 activation and DNA fragmentation in keratinocytes upon apoptotic stimuli. This could also explain the neuroprotective effect of Co Q through the increase of IFI 16.

In general, the similarity in the magnitude of the protective and therapeutic effects of CoQ and melatonin against PA neurotoxicity can be explained on the basis that both stimulate the expression of antioxidant and detoxification genes, acting in turn as a glutathione system enhancer. A further mechanism of protecting or enhancing cell survival by these two antioxidants lie in the control of damage and signaling function of mitochondria that involves decreased production of ROS. This could inturn; confirm the previously reported mechanism of PPA-induced neurotoxicity through oxidative stress-associated pathways [9,24,57].

High values of specificity, sensitivity and area under the curve (AUC) measured by ROC analysis suggested that the studied biomarkers could be used as predictive tools in testing the neurotoxic effects of PA and the potency of CoQ and melatonin in protecting or treating intoxicated rat pups. Among the measured parameters GABA and DNA damage markers were the most predictive recording high specificity and sensitivity and AUC of almost 1.

## Conclusion

Evidences suggest that nutritional deficiencies exacerbate pathological processes especially in developing children. However there have been very few intervention studies assessing the effects of specific nutrients on the prevention of cognitive decline. Based on the present study CoQ and melatonin can be suggested as nutritional supplements that might be helpful in the early intervention of neurodevelopmental disorders.

## Competing interests

The authors declare that they have no competing interests.

## Authors’ contributions

MA: Carried out the biochemical assays, AE: Designed the study and drafted the manuscript. LA: Co-drafted and revised the manuscript. All authors have read and approved the final manuscript.
